# Thermal stability of hepatitis E virus assessed by a molecular biological approach

**DOI:** 10.1186/1743-422X-8-487

**Published:** 2011-10-31

**Authors:** Anika Schielke, Matthias Filter, Bernd Appel, Reimar Johne

**Affiliations:** 1 Federal Institute for Risk Assessment, Diedersdorfer Weg 1, D-12277 Berlin, Germany; 2 Free University of Berlin, Faculty for Biology, Chemistry, Pharmacy, Takusstraße 3, D-14195 Berlin, Germany; 3 Robert Koch Institute, DGZ-Ring 1, D-13086 Berlin, Germany

**Keywords:** hepatitis E virus, stability, quantitative real-time RT-PCR, RNase A

## Abstract

**Background:**

Hepatitis E virus (HEV) is a pathogen of emerging concern in industrialized countries. The consumption of wild boar meat has been identified as one risk factor for autochthonous HEV infections. Only limited information is available about thermal stability of HEV, mainly due to the lack of rapid and efficient cell culture systems for measurement of HEV infectivity.

**Methods:**

A molecular biological method was implemented in order to distinguish disassembled from intact viral particles using RNase treatment followed by quantitative real-time RT-PCR. The method was applied to a wild boar liver suspension containing HEV genotype 3.

**Results:**

Time-course analyses indicated that the decline of protected RNA could be described by a biphasic model with an initial decrease followed by a stationary phase. The stationary phase was reached after 1 hour at 4°C, 3 days at 22°C and 7 days at 37°C with log reductions of 0.34, 0.45 and 1.24, respectively. Protected RNA was detectable until the end of the experiments at day 50 or 70. Heat exposure for 1 minute resulted in a log reduction of 0.48 at 70°C and increased with higher temperatures to 3.67 at 95°C. Although HEV infectivity titration by inoculation of the liver suspension onto three cell lines did not succeed, the results of the RNase-based method are in accordance with published cell culture-based data.

**Conclusions:**

Measurement of intact viral particles using the RNase-based method may provide data on the stability of RNA viruses when cell culture-based infectivity titrations are not efficient or not available. The method enables processing of large sample numbers and may be suitable to estimate stability of HEV in different types of food.

## Background

The hepatitis E virus (HEV) is classified as the only member of the genus Hepevirus, which is subdivided into four genotypes (GT) and the avian HEV strains. Recently, a novel hepatitis E-like virus has been detected in faeces of wild rats [1]. HEV is a non-enveloped icosahedral sphere of about 27 to 34 nm in diameter. Its genome consists of positive-sense single-stranded RNA of about 7.2 kb in length and contains three open reading frames (ORFs) [2-4]. ORF2 encodes the only capsid protein, forming the virus particle, whose atomic structure has been solved recently [5].

HEV is one of the leading causes of human acute viral hepatitis in Asia, the Middle East and Africa. In industrialized countries, this virus has recently been recognized as a pathogen of emerging concern [3,6]. Hepatitis E is characterized by a self-limiting jaundice of varying severity, which is hard to distinguish from other viral hepatitis infections and is often accompanied by unspecific symptoms like fever, headache and pain of the upper abdomen. Although the case-fatality rate of hepatitis E is low in the general population (0.5 to 3%), rates of up to 20% have been observed for pregnant women [7-9]. HEV is mainly transmitted via the faecal-oral route by contaminated drinking water or food. However, in industrialized countries, zoonotic transmission is suspected to be responsible for the increasing number of autochthonous cases, with wild boars and pigs regarded as the main virus reservoirs [10-13]. Reports on human hepatitis E cases after consumption of uncooked meat from wild boar or deer strengthened the hypothesis of a zoonotic transmission [14-16]. Recently, the consumption of wild boar meat has been identified as one risk factor for autochthonous HEV infections [6,9].

Only limited information is available about the heat stability of HEV, mainly due to the lack of an efficient, rapid and sensitive cell culture system. For all of the tissue culture systems for HEV published so far, a relatively high amount of virus is needed for successful infection, virus replication proceeds slowly and cytopathic effects are only rarely visible [4,17-19]. A first study showed that infection of A549 cells was prevented by heating of an HEV-containing cell suspension at 56°C for 30 minutes [20]. Using HepG2/C3A cells and an immunofluorescence assay for quantification of infective viruses, the thermal stability of HEV GT1 and GT2 strains was investigated in more detail: GT1 was nearly completely inactivated at temperatures between 56°C and 60°C for one hour, whereas GT2 turned out to be more resistant since only about 80% was inactivated at 60°C after one hour [21]. Time-course analyses showed that about 95% of GT1 was inactivated within the first 15 minutes at 56°C although remaining infectious virus was still detectable after one hour at this temperature [21]. Another study using PLC/PRF/5 cells showed that heating of a GT3-containing stool sample at 25°C or at 56°C for 30 minutes did not influence the infectivity in opposite to heating at 70°C or 95°C for ten minutes. At 95°C even one minute was sufficient to prevent the growth of the virus [19]. By monitoring of seroconversion of pigs experimentally inoculated with an HEV GT3-containing liver suspension it was shown that incubation at 56°C for one hour did not affect infectivity, whereas the suspension was no longer infective after heating at 191°C (internal temperature of 71°C) or above 100°C each for five minutes [22]. Yunoki et al. showed that proteins or magnesium may act as stabilizers, which can increase heat stability of HEV [23].

The objective of this study was to gain more detailed data about heat stability of HEV with regard to the kinetics of inactivation, the temperatures needed for complete inactivation and the influence of long-term storage at different temperatures. A wild boar liver suspension containing well characterized HEV GT3 [13] was selected for the experiments because of the mentioned assumptions about HEV transmission via wild boar meat to humans. Due to the challenges associated with an efficient cell culture system, a molecular biological approach was implemented. This approach employs an RNase treatment of the heated samples to remove any viral RNA released from disintegrated particles followed by quantification of the remaining RNA using real-time RT-PCR. Similar methods have been already published for differentiation between intact and disintegrated particles of hepatitis A virus, poliovirus, feline calicivirus and norovirus [24,25]. The novel approach may be suitable for application of larger sample numbers, e.g. to estimate the stability of HEV in different types of food, which is needed for further risk assessment of HEV transmission by food.

## Methods

### Viral stock preparation

A wild boar liver containing the genotype 3i HEV isolate wbGER27 [13], which had previously been shown to be closely related to an HEV isolate from a human hepatitis E case, was used in all experiments. The liver was thoroughly grinded in a mortar together with sterile sea sand and phosphate buffered saline (PBS, pH 7.4). After centrifugation at 3, 000 × g for 15 minutes, the supernatant was filtered through 0.2 μm and aliquots of the liver suspension were stored at -80°C until use. Analysis by quantitative real-time RT-PCR (see below) revealed 5 × 10^8 ^genome equivalents (GE) per ml in the liver suspension. Treatment with RNase A (see below) prior to RNA extraction resulted in 3 × 10^7 ^GE/ml corresponding to intact viral particles in the liver suspension.

### Exposure of the virus suspension to different temperatures

The liver suspension was thawed at room temperature and 100 μl aliquots were transferred to 1.5 ml Eppendorf-tubes, which were subsequently incubated in a heating block (Thermomixer comfort, Eppendorf, Hamburg, Germany) at temperatures between 56°C and 95°C for different time periods. For incubation at 4°C the aliquots were stored in the fridge (Liebherr, Ochsenhausen, Germany). A temperature of 37°C was ensured in an incubator (WTB Binder, Tuttlingen, Germany), while other aliquots were stored on the bench at room temperature (22°C). At each time-point, three replicates were removed and immediately frozen at -80°C. Aliquots frozen immediately without any RNase treatment and aliquots treated with RNase A but without previous heat treatment served as controls; water was used as negative control.

### RNase treatment and nucleic acid extraction

The RNase treatment is based on a protocol originally developed for noroviruses by Mormann et al., 2010 [24]. Briefly, 3.5 μl of RNase A (7000 units/ml, Qiagen, Hilden, Germany) diluted 1:10 with nuclease-free water was added to each sample and incubated at 37°C for one hour resulting in the degradation of free RNA released from disassembled viral particles during heat treatment. Thereafter, RNase A activity was inhibited by the addition of 18 units of QIAGEN RNase Inhibitor (Qiagen, Hilden, Germany) followed by an incubation at room temperature for 30 minutes. The RNA of the sample was isolated using the QIAamp Viral RNA Mini Kit (Qiagen, Hilden, Germany) according to the manufacturer's instructions. Finally, 50 units of Protector RNase Inhibitor (Roche Diagnostics, Mannheim, Germany) were added to the isolated RNA before an additional incubation step at room temperature for 30 minutes was applied. After this procedure, no residual RNase activity was detectable by the RNaseAlert Lab Test Kit (Applied Biosystems, Darmstadt, Germany). The isolated RNA was stored at -80°C until real-time RT-PCR analysis. After each experiment, the bench and the pipettes were thoroughly cleaned using RNase AWAY (Carl Roth, Karlsruhe, Germany) in order to eliminate any residual RNase.

### Quantitative real-time RT-PCR

For quantification of HEV RNA, in vitro transcribed RNA standards were prepared for generation of an external standard curve. Briefly, the complete region of ORF3, which partially overlaps with ORF2, was amplified by RT-PCR from the liver suspension using primers 5'-AGC GTC TAG AAT GAA TAA CAT GTY YTG TGC-3' and 5'-GAC ATC TAG ATC AAC GGC GCA GCC CCA GCT-3' and subsequently cloned into the vector pCR4 TOPO (Invitrogen, Karlsruhe, Germany). This vector contains M13 priming sites before and after a T7 promoter site attached to the insert. After confirmation of the sequence of the insert by DNA sequencing, a fragment containing the T7 promoter together with the ORF3 sequence was amplified by PCR with the universal M13 primers and the TaKaRa Ex Taq (Takara Bio Europe S.A.S., Saint-Germain-en-Laye, France). The resulting PCR product of 534 bp was purified using the QIAquick Gel Extraction Kit (Qiagen, Hilden, Germany). In vitro transcription was performed from the purified PCR product using the T7 MEGAscript Kit (Applied Biosystems, Darmstadt, Germany) according to the manufacturer's instructions. Thereafter, residual DNA was removed by the addition of 4 units TURBO™ DNase (Applied Biosystems, Darmstadt, Germany) followed by an incubation at 37°C for 30 minutes. The High Pure RNA Isolation Kit (Roche Diagnostics, Mannheim, Germany) was used for purification of the in vitro transcribed RNA essentially as recommended by the manufacturer; however, DNase I was not diluted before use. The absence of residual DNA was confirmed by a real-time PCR as described below, but by omitting reverse transcription. RNA was quantified by photometric measurement using the NanoDrop 1000 (Thermo Scientific, Wilmington, USA) and molecule numbers were calculated according to Fronhoffs et al., 2002 [26]. The real-time RT-PCR targeting a region of the ORF2/ORF3 junction of the HEV genome was performed as previously described [27] using the QuantiTect Probe RT-PCR Kit (Qiagen, Hilden, Germany). Briefly, the reaction mixture contains 10 μl of 2 × QuantiTect Probe RT-PCR Master Mix, 0.2 μl of Enzyme RT-Mix, the primers JVHEVF (5'-GGTGGTTTCTGGGGTGAC-3') and JVHEVR (5'-AGGGGTTGGTTGGATGAA-3') in a final concentration of 500 nM and the probe JVHEVP (5'-FAM-TGATTCTCAGCCCTTCGC-BHQ-3') in a final concentration of 100 nM filled up with nuclease-free water to a volume of 15 μl. Five μl of the sample or the RNA standards were added to the reaction mixture to a final volume of 20 μl. The reverse transcription proceeded at 50°C for 30 minutes, before an initial incubation at 95°C for 15 minutes followed by 45 cycles at 94°C for ten seconds, at 55°C for 20 seconds and at 72°C for one minute in an ABI PRISM 7500 cycler (Applied Biosystems, Foster City, USA). The threshold cycle (CT) was automatically calculated by the 7500 Software Version 2.0.1 (Applied Biosystems, Foster City, USA).

### Data analysis and statistics

The CT values of the tenfold serial dilutions from the in vitro transcribed RNA stock solution were determined and an external standard curve was generated by plotting the logarithmic concentration against these CT values. Each sample was analysed in triplicate. Linear regression analysis was then used for calculation of the number of GE present in the samples. Results designated as "undetermined" by the 7500 Software Version 2.0.1 (Applied Biosystems, Foster City, USA) were set equal to the detection limit of the PCR so that the log concentrations could be averaged and the standard deviation (SD) could be calculated. Descriptive statistical parameters (average, SD, 95%-confidence intervals) were calculated using Microsoft Excel 2003. The log reduction rate was defined as the logarithm of the quotient of the GE number of the heat treated sample and the GE number of the aliquot without previous heat treatment. The GE number of the liver sample without heat treatment was set as 100% for calculation of the inactivation rate expressed in percent. The data obtained from the long-term storage experiments were further analysed using the "Geeraerd and Van Impe Inactivation Model Fitting Tool" (GInaFiT) for Microsoft Excel [28] in order to fit a mathematic model to the data.

### Cell culture and immunofluorescence

Attempts to titrate infectious HEV were undertaken by inoculation of the liver homogenate preparations on the cell lines PLC/PRF/5, A549 and HepG2 followed by viral antigen detection by immunofluorescence. The cell lines were chosen according to published protocols for propagation of HEV [4,19-21]. All cell lines were grown in MEM supplemented with 10% foetal bovine serum (FBS), 1% L-glutamine, 1% non-essential amino acids and 0.5% gentamycine at 37°C and 5% CO_2 _atmosphere. The cell cultures were seeded on Lab-Tek glass chamber slides (Nalge Nunc International, Rochester, USA) and grown until 80% confluence. For infection, 100 μl of the wild boar liver suspension were inoculated onto the cells and incubated for one hour at room temperature. Thereafter, the virus suspension was replaced by medium without antibiotics and FBS and incubated at 34.5°C. After 24 h the supernatant was removed, medium containing antibiotics and 5% FBS was added and incubated for additional 5 days at 34.5°C. Thereafter, the cells were washed with PBS, fixed with acetone/methanol (1:1 v/v) at -20°C for 30 minutes and dried. A pig serum previously shown to contain HEV-GT3-specific antibodies using three different serological assays [10] was added at a 1:100 dilution in PBS and incubated at 37°C for one hour. After three washings with PBS, the secondary antibody FITC-conjugated anti-pig IgG (Sigma, Deisenhofen, Germany) was added at a 1:500 dilution and incubated at 37°C for 30 min. The slides were rinsed two times in PBS and one time in distilled water, dried and fluorescent cells were analyzed in a fluorescence microscope.

## Results

### Establishment of a quantitative real-time RT-PCR for HEV

For quantification of the HEV genome, RNA standards were constructed. After cloning of the complete HEV ORF3 into the vector pCR4 TOPO (Invitrogen, Karlsruhe, Germany), the insert was amplified by PCR with the universal M13 primers, purified and used for in vitro transcription. The resulting RNA concentration was determined to be 367.3 ng/μl with an OD_260/280 _ratio of 2.06 and an OD_260/230 _ratio of 2.31 indicating high purity of the RNA. The absence of residual DNA was confirmed by a real-time PCR without reverse transcription, which showed a negative PCR result. A linear correlation between CT values and the logarithmic concentration of serial dilutions of in vitro transcribed RNA was evident in the range between 66 and 6.6 × 10^9 ^GE per PCR assay, with a slope of -3.7 and a regression coefficient (R^2^) of 0.9968. The limit of detection of this real-time RT-PCR as determined by dilution series of the in vitro transcribed RNA was seven GE per PCR assay. Analysis of RNA isolated from the wild boar liver suspension used in the following experiments by the quantitative real-time RT-PCR revealed a concentration of 5 × 10^8 ^HEV GE per ml.

### Implementation of an RNase-based assay for measuring capsid-protected RNA

**Figure 1:**
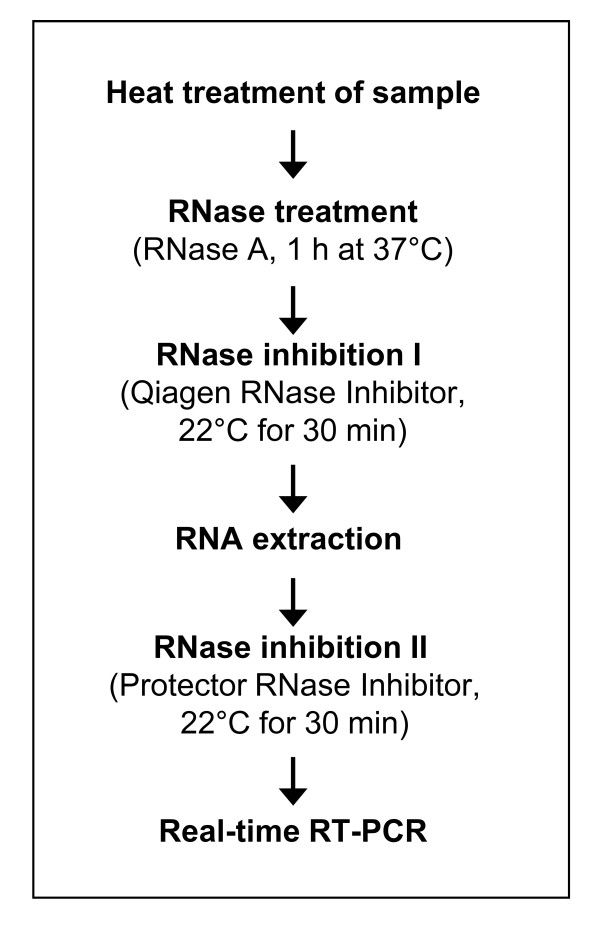
**Procedure of RNase treatment**. Schematic presentation of the procedure used for determination of heat stability of HEV using a quantitative detection method for capsid-protected RNA. After initial RNase treatment, the sample is subjected to two steps of RNase inhibition in order to remove any residual RNase activity.

PCR based methods are generally not suitable for differentiation between infectious and non-infectious viruses. The addition of an RNase may enable the differentiation between intact and disintegrated viral particles. The activity of the RNase, however, has to be inhibited before RNA extraction is performed and a final addition of a second RNase inhibitor should avoid any further RNA degradation after RNA isolation. For an overview of the applied procedure see Figure 1. In control experiments, 100 μl of in vitro transcribed RNA dilutions containing up to 1.3 × 10^7 ^GE or 100 μl of the wild boar liver suspension previously heated at 90°C for 30 minutes were subjected to the RNase treatment procedure. By a following real-time RT-PCR, no signal was detectable in these cases indicating that concentration and incubation conditions of the RNase A are sufficient to eliminate any free HEV-specific RNA present in the sample. In order to exclude a further RNA degradation after nucleic acid extraction, aliquots of the extracted RNA were tested by the RNaseAlert Lab Test Kit (Applied Biosystems, Darmstadt, Germany) demonstrating that no RNase activity was detectable. By application of the whole procedure to the wild boar liver suspension used in the following experiments, a concentration of 3 × 10^7 ^RNase-protected HEV GE per ml was determined.

### Inactivation by short-term heating

In order to enable comparison with published studies using tissue culture methods, the effect of short term heating on HEV was analysed at previously used temperature/time combinations. After an incubation at 56°C for 15 minutes, the amount of RNase-protected HEV RNA decreased remarkably as compared to the untreated control, with a reduction rate of 74.07% (-0.59 log_10_). The incubation of HEV at 56°C for 30 or 60 minutes, or at 60°C for 15 minutes, 30 minutes or 60 minutes led to reduction rates above 99% (-2.16 to -4.42 log_10_). These results are presented in Table 1 together with results of published cell culture-based studies. Incubation at 60°C for 90 minutes resulted in no detectable RNA (not shown).

**Table 1:** Comparison of data generated in this study and those published by other studies for HEV inactivation.

		Inactivation (tissue culture assay)				Reduction (RNase protection assay)
		
Temperature	Time	**Huang et al., 1999 **[20]	**Emerson et al., 2005 **[21]		**Tanaka et al., 2007 **[19]	This study
		GT1	GT1	GT2	GT3	GT3
56°C	15 min	ND	95%	ND	ND	74.07%
56°C	30 min	inactivated	99%	ND	not inactivated	99.99%
56°C	60 min	ND	99%	not inactivated	ND	99.90%
60°C	60 min	ND	96%	80%	ND	99.94%
95°C	1 min	ND	ND	ND	inactivated	99.98%

### Effect of long-term storage at different temperatures

**Figure 2:**
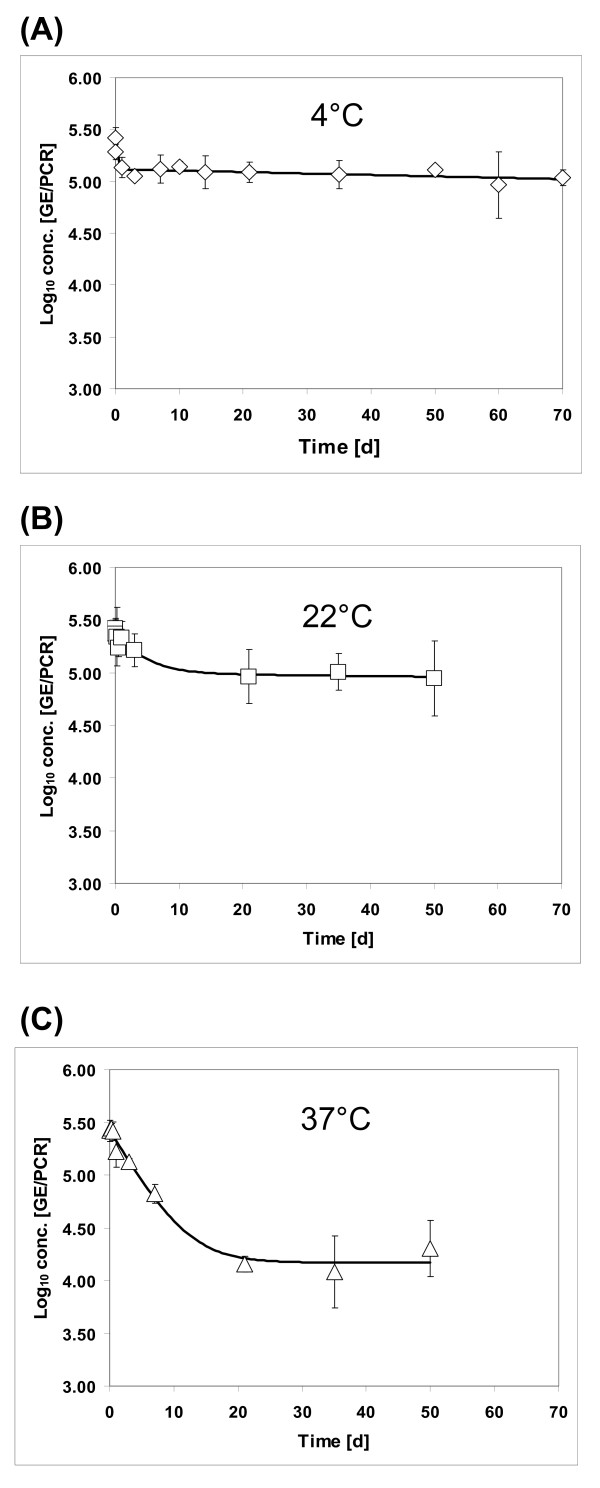
**Effect of long-term storage at different temperatures on HEV stability**. An HEV-containing liver suspension was incubated at 4°C (A), 22°C (B) or 37°C (C) for up to 70 days and protected RNA was quantified using real-time RT-PCR. The resulting data were used for fitting of a curve using a biphasic model according to Cerf, 1977 (29).

The influence of long-term storage conditions on the stability of HEV was tested by incubation of 100 μl aliquots of the liver suspension at 4°C, 22°C and 37°C. Protected HEV RNA was detectable at 22°C and 37°C as long as 50 days and at 4°C as long as 70 days, i.e. for the entire period of the experiments. The data of these inactivation trials could be fitted best by the biphasic model according to Cerf (1977) using GInaFiT for Microsoft Excel [28,29] (Figure 2A-C). As characteristic of this model, first a decrease of the intact viruses could be observed until a stationary phase is reached. For determination of the time-point of the change to the stationary phase the log concentrations of the last three data points were used to calculate the average stationary phase log concentration level. The associated 95%-confidence interval was then applied to classify the preceding measurements as "belonging" or "not belonging" to the stationary phase. If further data points had to be integrated into the calculation of the stationary phase average, the procedure was repeated until the first data point "not belonging" to the stationary phase was reached. Based on these calculations, the stationary phase is reached after one hour at 4°C resulting in a 0.34 log_10 _reduction and after three days at 22°C resulting in a 0.45 log_10 _reduction. At 37°C, the maximum reduction of 1.24 log_10 _was reached after seven days.

### Assessment of HEV stability at different temperatures for one minute

**Figure 3:**
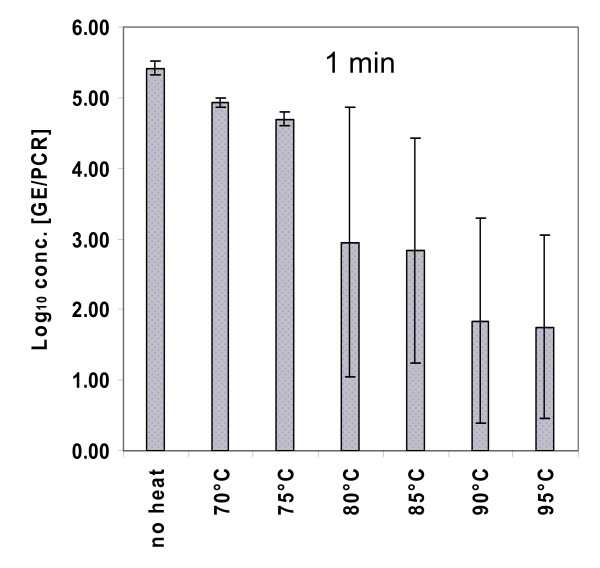
**Effect of short-term heating on HEV stability**. The HEV-containing liver suspension was heated at different temperatures for one minute, and protected RNA was quantified using real-time RT-PCR. The columns represent mean values from three independent tests.

The liver suspension (100 μl) was incubated at 70°C, 75°C, 80°C, 85°C, 90°C and 95°C each for one minute in triplicate (Figure 3). The exposure of HEV to 70°C for one minute led to an average log reduction of protected RNA of 0.48. At 75°C the reduction rate slightly increased (-0.72 log_10_). Incubation at 80°C and 85°C for one minute resulted in a 2.47-log_10 _and in a 2.58-log_10 _reduction, respectively, whereas the incubation at 90°C for one minute led to a 3.58-log_10 _reduction. Finally, the exposure of HEV to 95°C for one minute resulted in a log reduction of 3.67. The higher variability of the determined values at temperatures higher than 75°C as reflected by the high standard deviations (see Figure 3) coincided with the appearance of insoluble material precipitating from the organ suspensions at these temperatures.

### Inoculation of the virus preparation on cell cultures

In order to titrate infectivity of the virus preparation used in the RNase-based assay, attempts were undertaken to demonstrate virus infection in cell cultures. Three types of monolayer cell cultures were inoculated with the liver suspension and the presence of viral antigen in the cells was analyzed by immunofluorescence at 6 days after inoculation. For PLC/PRF/5 cells, fluorescence could not be quantified due to extensive background staining as assessed by comparison with a negative control. In HepG2 cells and in A549 cells, isolated cells with a stronger staining than observed in the negative control could be detected in the slides inoculated with the native liver suspension (not shown). However, the number of stained cells varied remarkably and fluorescent cells were not detected in all replicates inoculated with this preparation. A long-term infection trial was performed by inoculation of the native liver suspension onto the three cell lines followed by incubation at 34.5°C for 30 days. Testing of aliquots of the culture supernatants using real-time RT-PCR revealed constantly decreasing amounts of the HEV genome over the whole duration of the experiment (data not shown).

## Discussion

The increasing number of recorded hepatitis E cases led to the recognition of HEV as an emerging pathogen in industrialized countries [30]. Since many of these infections cannot be explained by importation of HEV infections from endemic regions, attention has been drawn to alternative routes of transmission. Due to the detection of HEV strains closely related to human HEV in wild boars and pigs, these animals are regarded as reservoirs of this virus [13,31,32]. By a case control study performed in Germany and by investigations on food-borne hepatitis E outbreaks in Japan, a transmission route via the consumption of wild boar meat is supported [9,14,15,33,34]. In order to provide guidelines for prevention of such food-borne transmissions, studies determining inactivation kinetics of HEV exposed to heating and long-term storage are currently needed.

Most of the previous studies assessing thermal stability of HEV are based on different cell culture systems for the detection of infectious HEV particles [19-21,23]; and one study used a swine bioassay [22]. Because the used cell culture techniques and animal experiments are sophisticated, time-consuming and relay on special facilities, studies with larger sample numbers could not be performed and only some selected temperature/time combinations have been tested so far. Isolation and propagation of HEV in tissue culture is difficult and dependent on the amount of virus used for inoculation [4,19,35]. Very recently, an HEV variant, which showed a more rapid growth in cell culture, has been isolated from a chronically infected patient [36]; however, the significance of this finding has to be investigated further. In almost all studies performing animal experiments, pigs are infected intravenously with HEV although this kind of inoculation does not reflect the natural route of transmission. However, pigs infected orally with HEV need larger virus amounts [37-39]. Therefore, low amounts of infectious virus present in the sample after heat inactivation may not be detected using these test systems. As no reliable cultivable surrogate for HEV is known until now, other techniques may help to estimate heat stability of this virus.

Recently, a molecular biological approach has been applied successfully for the assessment of the stability of noroviruses during technical processes [24]. Similar approaches have been used for stability studies with hepatitis A virus, poliovirus type 1 and feline calicivirus [25,40]. In these approaches, remaining 'intact' viral particles present after treatment are identified by their ability to protect the viral RNA genome from degradation. Although these 'intact' viruses are per se not necessarily infectious, a direct correlation between detection of protected RNA and detection of cell-culture infectious virus has been described [41]. The major advantage of this molecular biological method is that it can be implemented by any laboratory proficient in molecular biological techniques and that several temperature/time combinations can be tested. This is especially important when information about the stability of food- or waterborne pathogens is limited and an efficient cell culture method or animal model does not exist. The described method may also be relevant for routine safety controls of suspicious foods, whereas cell culture-based methods may not be applicable for routine diagnostic. For risk assessment analyses it might be advantageous that molecular biological techniques tend to overestimate the stability of the viruses since all particles are detected, which protect the viral RNA from degradation, even if these particles may no longer be infectious due to conformational changes on the surface of the capsid protein, whereas an underestimation of the viral load may have serious consequences.

The molecular biological method described here was attempted to be validated by using cell culture systems described earlier [4,19-21]. However, although some fluorescent cells were detected at 6 days after inoculation of the native liver suspension, a quantification of infectious HEV particles in the preparation and a convincing demonstration of virus replication in the cell cultures were not possible. The distinct reasons for these findings are not known, but as already described, propagation of HEV in cell culture is difficult and relies on a number of prerequisites, which may have missed in these experiments. First, all of the described successful propagation trials of HEV in cell cultures were performed with inocula containing very high HEV titres [4,19-21]. The amount of infectious HEV present in our sample might be too low. Second, only inoculation of serum and faecal samples in cell cultures has been described to result in successfully HEV propagation so far [4,17-19]. It might be speculated that preparations of liver tissue contain only a low proportion of HEV which is able to infect cell cultures. Third, it is not known if the distinct HEV strain present in our liver suspension is capable of efficient propagation in the used cell lines. The used strain was of the GT3 subtype i [13], for which propagation in cell cultures has not been described so far.

As a direct validation of our results using cell culture experiments could not be done, we used published data on HEV stability derived from cell culture experiments and experimental animal inoculations for comparison. As shown in Table 1, the results of our study using the molecular biological approach are generally in accordance with that of the published studies. For example, inactivation rates between 99% and 100% have been reported for HEV after incubation at 56°C for 30 minutes [20,21]. Using the same conditions, we determined a reduction rate of 99.99% (-4.42 log_10_). Heating of HEV at 56°C for 15 minutes resulted in an inactivation rate of 95% [21], which is comparable with the reduction rate of 74% (-0.59 log_10_) determined in our approach. At 60°C for one hour, inactivation rates of 96% and 80% were described for HEV GT1 and GT2, respectively [21], whereas in our experiments, 99.94% (-3.25 log_10_) of HEV GT3 was inactivated under these conditions. It is also evident from this comparison, that additional factors like the used genotype, the used virus amount and the type of cell cultures may further influence the result.

The data of our long-term storage trials generated with the molecular biological approach could be fitted best by a biphasic model according to Cerf, 1977 [28,29]. The major characteristics of this model are an initial decrease of intact viruses followed by a stationary phase, where the virus is inactivated only very slowly. The same model has been successfully applied previously for inactivation curves of foot-and-mouth disease viruses [42]. Generally, relative low reduction rates were recorded for HEV at 4°C, 22°C and 37°C, indicating that HEV is able to survive for a long time in the environment. The maximum reduction of 1.24 log_10 _was reached after seven days at 37°C. Thereafter, the viral load remained constant for several weeks on a relatively high level. Similar inactivation curves showing a tailing in the logarithmic plot were reported for murine norovirus, feline calicivirus and poliovirus exposed to different temperature and pressure conditions [43-45]. Since an adaptation of the viruses to the different environmental conditions is not possible, the stationary phase may reflect the heterogeneous sensitivities of the virus particles to different temperatures [43].

For short-term heating, our data show that exposure of HEV to 70°C for one minute resulted only in a log reduction of 0.48. According to Koopmans and Duizer (2004), the risk of infection for the consumer after cooking contaminated meat could be categorized as low or negligible by a log reduction of 3 or 4, respectively [46]. Based on our data, a log reduction of 3 could be only achieved after heating at 90°C for one minute, and incubation at 95°C for one minute was necessary for a log reduction of about 4.

## Conclusions

In summary, this study provides novel data on the thermal stability of HEV using a molecular biological approach, which is able to differentiate between intact and disintegrated virus particles. Although these data show that HEV is considerable sensitive to heat, residual infectious virus has to be expected after most short-term heating procedures of meat, which may explain the documented cases of hepatitis E after consumption of grilled wild boar meat [14-16]. Other temperature/time combinations may be tested in further studies in order to provide a safe procedure for preparation of meat without the risk of HEV infection. As the type of food and its specific processing may also influence the stability of HEV, many different parameters have to be tested, which may be more easily analysed using the molecular biological approach.

## List of abbreviations

bp: base pairs; CT: threshold cycle; DNA: deoxyribonucleic acid; FBS: foetal bovine serum; FITC: fluorescein isothiocyanate; GE: genome equivalents; GT: genotype; HEV: hepatitis E virus; IgG: Immunoglobulin G; kb: kilobases; MEM: Minimum Essential Medium; OD: optical density; ORF: open reading frame; PBS: phosphate buffered saline; PCR: polymerase chain reaction; R^2^: regression coefficient; RNA: ribonucleic acid; RT-PCR: reverse transcription polymerase chain reaction; SD: standard deviation.

## Competing interests

The authors declare that they have no competing interests.

## Authors' contributions

AS carried out the molecular biological methods and drafted the manuscript. MF conducted the statistical analyses and helped by critical reading of the manuscript. BA was included in drafting the manuscript and critical reading. RJ was responsible for the design and conception of the study and helped by drafting and critical reading of the manuscript. All authors read and approved the final manuscript.
